# How Good It Would Be to Turn Back Time: Adult Attachment and Perfectionism in Mothers and Their Relationships with the Processes of Parental Identity Formation

**DOI:** 10.5334/pb.492

**Published:** 2020-02-28

**Authors:** Konrad Piotrowski

**Affiliations:** 1 Institute of Psychology, SWPS University, Poznań, PL

**Keywords:** perfectionism, adult attachment, identity, parental identity, U-MICS

## Abstract

Parental identity formation may be a factor of the utmost importance in helping us to understand the mechanisms of adaptation to parenthood. However, our knowledge regarding the processes involved in the development of parental identity is very limited. In the present study the relationships between three dimensions of parental identity (commitment, in-depth exploration, reconsideration of commitment), and two trait-like characteristics that determine the quality of family life, i.e. romantic adult attachment and perfectionism were analyzed. 206 mothers aged 22 to 40 participated in the study (*M* = 33.33, *SD* = 3.68). The results revealed that a high level in anxious attachment, avoidant attachment and maladaptive aspects of perfectionism (other-oriented and socially-prescribed perfectionism) positively correlate with a low level of parental identity commitment and a high level of reconsideration of parental commitment. Regression analysis revealed that especially attachment-related anxiety and other-oriented perfectionism can be treated as independent, specific predictors of an increased crisis of parental identity.

## Introduction

One of the key developmental processes, beginning with the adolescence period throughout adulthood, is formation of identity, understood as development of a relatively stable self-definition, by undertaking strong commitment in important domains (Vignoles, Schwartz, & Luyckx, 2011). Well-developed identity contributes to easier decision making, provides motivation to achieve set goals, supports coping with emotions and contributes to the overall quality of life (Kroger & Marcia, 2011). Identity development, however, moves across various trajectories. Such characteristics as anxiety, indecision, depression which may hinder the process of making important decisions or increase doubts and difficulties at a later time, after making identity decisions, contributing to persistence of the identity crisis (Crocetti, Rubini, & Meeus, 2008; Crocetti, Klimstra, Keijsers, Hale, & Meeus, 2009; Luyckx et al., 2008; Piotrowski, 2019).

The presented study was devoted to formation of parental identity (Fadjukoff et al., 2016; Piotrowski, 2018), which is an identity domain that is rarely included in contemporary, processual research on identity (Luyckx, Schwartz et al., 2008; Crocetti, et al., 2008). Yet, parental identity formation may help us better understand the mechanisms of adaptation to parenthood. The transition to parenthood is a life-changing process that has a significant impact on the subsequent development of a parent and a child. Although people who have become parents often experience positive changes (Chen, Enright, & Tung, 2016), difficulties and burdens are also typical parental experiences that increase the risk of later maladjustment (Hansen, 2012; Pollmann-Schult, 2014). Difficulties in the transition to parenthood can result in mental health problems like depression and anxiety, and can lead to low satisfaction with life (Perren et al., 2005). Taking into account the recently stressed role of identity development in the occurrence of psychopathology (Klimstra & Denissen, 2017), it is reasonable to suggest that parental identity development can be a crucial factor that could help us better understand the adaptation to parenthood and parental psychopathology. However, our knowledge regarding the processes involved in the formation of parental identity is very limited.

### The three-dimensional conceptualization of parental identity formation

Delmore-Ko (2000) defines parental identity as a dynamic, mental representation of oneself as a parent. Recently, Piotrowski (2018) proposed to include the parental domain in the Meeus-Crocetti processual identity model (Crocetti, Rubini, & Meeus, 2008) in order to better understand this internal dynamic of the parental identity. According to this proposal, when an individual becomes a parent, there is a process of greater or lesser identity *commitment* and identification with the parental role, which manifests itself in the level of satisfaction and self-confidence in performing this role. Identity commitment in the parental domain is usually accompanied and fostering by *in-depth exploration*, which is expressed in search for information about the child and about parenting. However, as parenthood can be a demanding and stressful experience, leading in some cases to low adaptation and satisfaction (Delmore-Ko, Pancer, Hunsberger, & Pratt, 2000; Harwood, McLean, & Durkin, 2007), a parent may also start to regret becoming one (Donath, 2015) and fantasize that it would be better not to be a parent at all. In Piotrowski’s conceptualization (2018), this is how *reconsideration of commitment* is understood, being the third parental identity dimension in this model and an indicator of a parental identity crisis. In his research, Piotrowski (2018) showed that identity commitment in the parental domain is positively related to general life satisfaction, to vocational identity development, and to a general sense of identity, whereas reconsideration of commitment in the parental domain is negatively related to the quality of life and general identity development. In his research, it also turned out that in the group of mothers, a high level of trait-anxiety (Spielberger et al., 1983) and the diffuse-avoidant identity style (avoiding attitude towards identity decisions; Berzonsky, 1989), were significantly positively related to difficulties in developing a stable parental identity. This suggests the importance of parent personality characteristics for the dynamic of identity processes in the parental domain.

The links between the sense of identity and higher-order personality factors such as the Big Five are well-established (Klimstra, Luyckx, Goossens, Teppers, & De Fruyt, 2013; Lounsbury, Levy, Leong, & Gibson, 2007). However, there still is a need to study the role of more narrow and specific lower-order personality characteristics (McAdams & Pals, 2006). As parental identity, along with relational identity (Crocetti, Rubini, & Meeus, 2008), is a crucial domain of the sense of identity in a family context, it is especially important to establish its relationships with factors that strongly influence interactions with family members (both a partner and a child) and are broadly related to the adjustment, mental health, and the quality of life.

In the presented study, it was decided to assess the role of two relatively stable and related to each other trait-like characteristics whose links with the quality of close relationships within a family and the psychopathology risk are well-known: romantic adult attachment (Hazan & Shaver, 1987) and trait perfectionism (Hewitt & Flett, 1991). The present study is the first test of the links between those characteristics and parental identity formation.

### Attachment-related emotions in a family context

Adult attachment theory focuses on individual differences regarding relatively stable (but susceptible to change as an effect of important experiences) patterns of functioning in close interpersonal relationships (Mikulincer & Shaver, 2007). The development of attachment patterns begins in early childhood in the relationship with caregivers (early attachment), however, during adolescence and adulthood, the role of attachment figures begins to be taken up by peers, mainly partners in romantic relationships (Hazan & Shaver, 1987). Although individuals develop multiple mental representations of attachment such as a general attachment representation and representations of a few specific, important relationships, e.g. with a parent, a peer, etc., in adulthood it is romantic attachment (i.e. mental representation of attachment to a romantic partner) that has the strongest influence on adjustment, especially in relational context (Cozzarelli, Hoekstra, & Bylsma, 2000). According to this observation, the adult romantic attachment was also studied in the present project focused on family environment. The quality of attachment to a partner is usually assessed through the prism of two key dimensions: attachment-related *anxiety* (fear of abandonment, feeling of being unimportant for a partner) and attachment-related *avoidance* (feeling of discomfort in a situation of high intimacy, reluctance to open up to the partner and rely on him; Mikulincer & Shaver, 2007). Persons characterized by a low level of these two tendencies (securely attached) are characterized by the most satisfying functioning, both on intrapersonal and interpersonal levels. In turn, high attachment anxiety is related, among other things, to the desire to control the partner and limit his autonomy, the pursuit of excessive closeness, frequent expression of negative emotions, hypersensitivity to lack of attention. High avoidance in the relationship is related to a more pragmatic rather than romantic approach to relationships, unwillingness to respond to the partner’s signals expressing closeness, negating and suppressing negative emotions.

Romantic adult attachment was proved to be one of the main indicators of the quality of an intimate relationship (Mikulincer & Shaver, 2007). Both attachment anxiety and avoidance correlate negatively with satisfaction from an intimate relationship (Mikulincer & Shaver, 2007) but adult attachment patterns influence not only interactions with a romantic partner but also with a child. Secure adult attachment representation helps a caregiver to be more open to a child’s needs, promote higher responsiveness and more optimal parenting styles (Feeney & Woodhouse, 2016; Millings, Walsh, Hepper, & O’Brien, 2013). Parents with lower attachment anxiety and avoidance are also more satisfied with parenting (Mikulincer & Shaver, 2013).

Development of an attachment representation during interactions with caregivers in childhood and with romantic partners and friends in adolescence and adulthood is also related to changes in other areas of psychological functioning (Mikulincer & Shaver, 2016). Hewitt, Flett, and Mikail (2017) suggest that disruptions in attachment can sometimes lead to development of perfectionism which in this context is seen as a form of a defense mechanism aimed at regulation of self-esteem and at garnering acceptance and protecting against rejection thanks to ‘being perfect’ in everything (see Ko, Hewitt, Cox, Flett, & Chen, 2019). However, it needs to be further investigated.

### Perfectionism and identity formation

Perfectionism is defined as a strong pursuit of perfection and flawlessness, accompanied by an excessive tendency to self-criticism and fear of judgment (Frost et al., 1990, Hewitt & Flett, 1991). This characteristic, although susceptible to change and often motivated by external factors (Ashbaugh, Antony, Liss, Summerfeldt, McCabe, & Swinson, 2007; Suh, Sohn, Kim, & Lee, 2019), is often observed as having a tendency to being relatively stable in time (Rice & Aldea, 2006; Sherry, Richards, Sherry, & Stewart, 2014). The authors of the most popular current model of perfectionism, Hewitt and Flett (1991), perceive perfectionism as personality characteristic with three main manifestations: *self-oriented perfectionism* (the expectation of self-perfection in everything that is done), *other-oriented perfectionism* (strong expectation from others, especially significant ones, that they will aim for perfection and not make mistakes), and *socially prescribed perfectionism* (the belief of an individual that other people, especially important and close ones, expect them to be flawless and perfect). Similarly as in the case of attachment-related patterns, in terms of perfectionism, its roots can be traced back to childhood, although development of this characteristic may also take place at a later time (Hewitt, Flett, & Mikail, 2017).

Self-oriented perfectionism is often seen as the most adaptive facet of perfectionism, while socially-prescribed and other-oriented perfectionism are seen as predictors of a broad range of personal and social difficulties (Stoeber & Otto, 2006). Self-oriented perfectionism is connected with high consciousness, high achievement motivation, and with more satisfaction with close relationships (Stoeber & Otto, 2006). Self-oriented perfectionism positively correlates also with the most optimal, authoritative parenting style (Flett, Hewitt, & Singer, 1995; Snell et al. 2005), while maladaptive forms of perfectionism, i.e. socially-prescribed and other-oriented perfectionism, are more related to authoritarian, overprotective, and controlling parenting (Frost, Lahart, Rosenblate, 1991; Gong, Fletcher, & Bolin, 2015; Smith et al., 2017).

In line with the idea of the close links between attachment disruptions and maladaptive perfectionism (Hewitt, Flett, & Mikail, 2017), Wei et al. (2004) showed that maladaptive forms of perfectionism (they studied constructs that were similar to socially-prescribed perfectionism from the Hewitt and Flett’s model) are positively correlated with both romantic attachment anxiety and avoidance. They also observed that such indicators of the maladaptive aspects of perfectionism like concern over mistakes, doubts about one’s action, perceiving oneself as failing to meet personal standards for performance (Frost et al., 1990) mediate between romantic attachment patterns and depression in students. The studies on the attachment-perfectionism link clearly show that it is indeed a socially-prescribed aspect of perfectionism that is the main correlate of insecure attachment (Chen, Hewitt, & Flett, 2015).

Another aspect of perfectionism that is strongly related to difficulties in social interactions is other-oriented perfectionism that is seen as the ‘dark side’ of perfectionism. Stoeber (2014, 2015) showed that people who expect perfection from others differ from those who expect perfection mainly from themselves. They are more narcissistic, psychopathic, machiavellistic, less socially oriented and focused more on control and power. The romantic relationships that they create are full of tensions and conflicts (Shea, Slaney, & Rice, 2006).

### Attachment, perfectionism, and identity

While we know that attachment and perfectionism, especially socially-prescribed perfectionism, are related to each other and that both strongly influence family life, their links with identity development are less known, especially in regard to perfectionism. The varied attachment patterns may underlie different capacities for exploration and commitment in adolescence and adulthood. Secure attachment representation gives people a general sense of security and purpose while relationships with others based on a sense of security are a great source of comfort and support that may be a help in resolving the identity crisis (Årseth et al., 2009). The last meta-analysis of research on the relation of attachment (mostly romantic attachment) and identity presented by Årseth et al. (2009) leads to the conclusion that high attachment anxiety and high attachment avoidance are indeed associated with difficulties in forming a stable identity during adolescence and early adulthood and that both of these attachment-related emotions are related to identity confusion. However, Årseth et al., (2009) observed that those relationships are not strong. From a perspective of the Meeus-Crocetti identity model (Crocetti et al., 2008) it would translate into lower commitment and in-depth exploration, and higher reconsideration of commitment in individuals high on attachment anxiety and avoidance.

The studies on the relation of perfectionism and identity are still rare. Only two studies carried out so far were directly devoted to this issue. Luyckx, Soenens et al., (2008) observed that striving for perfection (similar to self-oriented perfectionism in the Hewitt and Flett’s model) in late adolescence is associated with positive identity development and correlate with stronger identity commitment, higher identification with commitments undertaken, and higher exploration, while perfectionistic doubts, fear of criticism and negative evaluation of others (similar to socially-prescribed perfectionism) are associated with difficulties with commitment making. In the second study, Piotrowski (2019) confirmed the Luyckx’s results and showed that such emotional and cognitive characteristics associated with perfectionism as indecisiveness, shame proneness, worry, and rumination are important mediators of the perfectionism-identity link. Other-oriented perfectionism has not been previously studied in the context of identity development. Neither attachment nor perfectionism has previously been studied in the context of processes of identity formation in the parental domain. The present study was aimed at fulfilling these gaps.

## Research problems and hypothesis

The main aim of the presented study was to assess for the first time the links between adult attachment-related emotions, different facets of perfectionism, and identity processes in the parental domain. Parental identity was conceptualized according to the three-dimensional identity model (Crocetti, Rubini, & Meeus, 2008) adapted by Piotrowski (2018) to the parental domain.

As both romantic attachment-anxiety and attachment-avoidance are positively associated with negative emotional interactions, with many tensions in a family, worse coping with negative emotions and stress, and with lower satisfaction with parenting (Jones, Cassidy, & Shaver, 2015; Mikulincer & Shaver, 2013), it was assumed that those attachment dimensions may also be positively related to difficulties in parental identity formation. In line with this suggestion, it was hypothesized that attachment anxiety and avoidance would negatively correlate with parental identity commitment and in-depth exploration and positively with reconsideration of commitment in the parental domain.

With regard to perfectionism, it was expected that self-oriented perfectionism would be positively associated with commitment and in-depth exploration, and negatively with reconsideration of commitment while socially-prescribed perfectionism would be related negatively to commitment and in-depth exploration and positively to reconsideration of commitment (see Luyckx, Soenens et al., 2008). It would be in line with perceiving self-oriented perfectionism as an adaptive and socially-prescribed perfectionism as a maladaptive form of perfectionism (Stoeber & Otto, 2006). Relationship between other-oriented perfectionism and identity development has not been studied so far, however, this dimension of perfectionism positively correlates with antisocial behavior and concentration on oneself and one’s own needs (Stoeber, 2014, 2015), which may be contradictory to fulfilling the role of a parent. Accordingly, it was assumed that it would positively correlate with the severity of parental identity crisis, and thus with higher reconsideration of commitment and with lower identity commitment.

## Materials and methods

### Participants

Studies suggest that the role of a parent is more salient for women (Kerpelman & Schvaneveldt, 1999; Williams & Kelly, 2005) and that women experience higher parenting stress than men (Simon, 1992; Hildingsson & Thomas, 2013). Recently, Roskam, Brianda, and Mikolajczak (2018) and Sorkkila and Aunola (2019) showed that mothers may also be more prone than fathers to parental burnout. In line with those results, it was decided to study the group of mothers in the present project. However, in the future, it would be necessary to assess gender differences in parental identity as we know that both mothers and fathers feel stressed as parents (Roskam, Brianda, & Mikolajczak, 2018).

206 mothers in the period of emerging and early adulthood participated in the study (aged 22 to 40 years; *M* = 33.33, *SD* = 3.67). Participants had from one (47%) to four children (1%) whose age was from 6 months to 14 years (*M* = 4.40, *SD* = 2.88). At the time of the study, all mothers were in a romantic relationship (it was not controlled whether the current partner is the father of the child/children). The average duration of a relationship, in years, was *M* = 9.88, *SD* = 4.62. All participants of the study were Polish. Informed consent was obtained from all individual participants included in the study. The present study (a part of the research project entitled ‘Parental identity formation in early and middle adulthood’) was approved by the Ethics Committee at the author’s institution.

### Measures

*Parental identity.* The Utrecht-Management of Identity Commitments Scale (U-MICS; Crocetti, Rubini, & Meeus, 2008) in a version adapted to measure three identity processes in the parental domain was applied (Piotrowski, 2018): commitment (5 items, e.g., *Being a parent gives me security in life*), in-depth exploration (5 items, e.g., *I make a lot of effort to keep finding out new things about my child/children*), reconsideration of commitment (3 items, e.g., *I often think it would have been better not to have had any children*). In earlier studies by Piotrowski (2018), reconsideration of commitment subscale consisted of two items, which could lead to lower reliability of this indicator. In order to increase the psychometric value of the questionnaire, it was decided to add a third item to this subscale. Thanks to this, the version used in this study (see Appendixes) had the same number of items as all other U-MICS versions (see Crocetti, Rubini, & Meeus, 2008). The 13-item scale was characterized by a hypothesized, three-dimensional structure, *X^2^* (62) = 126.96, CFI = .95, RMSEA = .07, and acceptable reliability measured by Cronbach’s alpha coefficient, respectively: .87, .69, .88.

*Adult attachment.* The Experiences in Close Relationships – Relationship Structures (ECR-RS; Fraley, Heffernan, Vicary, & Brumbaugh, 2011; Polish adaptation: Marszał, 2015) was used to assess the dimensions of attachment in relation to the partner. ECR-RS consists of 9 items that allow to measure: attachment-related avoidance (6 items; e.g., *I don’t feel comfortable opening up to my partner*) and attachment-related anxiety (3 items; e.g., *I’m afraid that my partner may abandon me*). Cronbach’s alphas were .90 and .86, respectively. The factor structure of the scale was not ideal but acceptable, *X*^2^ (23) = 73.11, CFI = .96, RMSEA = .10.

*Perfectionism.* The shortened 15-item version of the Hewitt Multidimensional Perfectionistic Scale (HMPS, Hewitt, Habke, Lee-Baggley, Sherry, & Flett, 2008; Stoeber, 2016) was used to measure the three facets of perfectionism described in the Hewitt and Flett model (1991): self-oriented perfectionism (5 items, e.g., *One of my goals is to be perfect in everything I do*), other-oriented perfectionism (5 items, e.g., *If I ask someone to do something, I expect it to be done flawlessly*) and socially prescribed perfectionism (5 items, e.g., *People expect nothing less than perfection from me*). The scale has been translated into Polish using the back-translation procedure. The author of the article directed the translation process. The present study was the first use of this questionnaire in a Polish sample. The scale was characterized by a good factor structure, *X*^2^ (86) = 244.34, CFI = .91, RMSEA = .09. Cronbach’s alphas were, respectively, .87, .78 and .83.

### Procedure and analytic strategy

Data were collected online with the use of electronic versions of the measures. There was only one measurement point. In order to reach participants, the researcher availed himself of his private contacts and the snowball method. The participants were informed about the aim of the research and signed a consent of participation. In the instructions, there was a request to give answers to all the questions included in the questionnaire. It was explained that this would be helpful in further data analysis.

When analyzing the dataset, firstly, descriptive statistics and Pearson’s correlations between variables were calculated. In the next step, specific relationships between particular dimensions of parental identity and other variables were analyzed. Three regression analyses were carried out, in which identity processes in the parental domain (commitment, in-depth exploration, and reconsideration of commitment) were treated as criterion variables and the dimensions of attachment and perfectionism as predictors. All analyses were performed with the use of SPSS 25 software. Treating attachment and perfectionism as predictors of parental identity in the regression was mostly based on theoretical assumptions. As roots of attachment and perfectionism are usually grounded in early development it can be assumed that in real life those characteristics can shape parental identity development after childbirth. It, of course, does not preclude further mutual relationships between those characteristics. However, as the present study was cross-sectional we could have only assessed if mothers who differ on attachment and perfectionism experience, at the same time, more difficulties with the formation of a stable parental identity. Regression analysis also allows observation of specific relationships between predictors and dependent variables that was also seen as important at the early stage of studies on parental identity processes.

## Results

### Descriptive statistics and correlational analysis

Descriptive statistics and correlations between analyzed variables are presented in Table 1. Identity commitment correlated negatively with attachment anxiety. In-depth exploration was, in turn, positively associated with the level of self-oriented perfectionism. The most significant relationships occurred in the case of reconsideration of commitment, which turned out to be positively correlated with avoidance and anxiety in a romantic relationship and with other-oriented perfectionism as well as socially-prescribed perfectionism. There was also a significant, positive correlation between attachment anxiety and avoidance and socially prescribed perfectionism. Correlations between parental identity, attachment, and perfectionism were weak to moderate.

**Table 1:** Descriptive Statistics and Correlations Between Analyzed Variables.

	*M*	*SD*	2	3	4	5	6	7	8

1. Commitment	3.65	.84	.20**	–.56***	–.14	–.19**	.04	–.02	–.03
2. In-depth exploration	4.17	.50	–	–.10	–.09	–.01	.14*	.10	.10
3. Reconsideration of commitment	1.43	.71		–	.19**	.29***	.06	.20**	.15*
4. Avoidance	2.04	1.26			–	.52***	–.02	.09	.21**
5. Anxiety	2.00	1.45				–	.06	.07	.21**
6. Self-oriented perfectionism	5.16	1.40					–	.54***	.42**
7. Other-oriented perfectionism	4.01	1.35						–	.48***
8. Socially-prescribed perfectionism	3.49	1.47							–

* *p* < .05, ** *p* < .01, *** *p* < .001.

Correlations between dimensions of parental identity and mother’s age, number of children, age of children (in case of having more than one child, their average age was used), duration of the current romantic relationship were also analyzed. A significant relationship was observed only in one case, the level of identity commitment was negatively correlated with the mother’s age, *r* = –.16, *p* <.05.

### Adult attachment and perfectionism as predictors of parental identity processes

Due to the significant correlation between the mother’s age and identity commitment, age was controlled in all regression models. The results of regression analysis are shown in Table 2.

**Table 2:** The Results of the Regression Analyses.

		Commitment	In-depth exploration	Reconsideration of commitment

Predictors		*β*	*β*	*β*

Step 1	Age	–.16*	–.12	.04
		*R*^2^ = .03 *F =* 5.57*	*R*^2^ = .02 *F =* 3.64 *ns*	*R*^2^ = <0.1 *F =* .29 *ns*
Step 2	Age	–.16***	–.11	.03
	Avoidance	–.03	–.11	.03
	Anxiety	–.18*	.03	.26**
	SOP	.06	.09	–.08
	OOP	–.03	.03	.22**
	SPP	.01	.06	.01
		*R*^2^ = .07	*R*^2^ = .05	*R*^2^ = .12
		*F =* 2.39*	*F =* 1.64 *ns*	*F =* 4.67***
		Δ*R*^2^ = .04	Δ*R*^2^ = .03	Δ*R*^2^ = .12
		Δ*F* = 1.73 *ns*	Δ*F* = 1.19 *ns*	Δ*F* = 5.54***

Note: In the table standardized beta coefficients are presented. SOP – self-oriented perfectionism, OOP – other-oriented perfectionism, SPP – socially prescribed perfectionism. * *p* < .05, ** *p* < .01, *** *p* < .001.

While taking into account all predictors in the regression model, identity commitment in the parental domain turned out to be significantly, negatively related only to the age (*β* = –.16) and to the degree of the mother›s attachment anxiety (*β* = –.17). In the case of in-depth exploration, no significant relationships were found with attachment and perfectionism. It turned out, however, that the degree of reconsideration of commitment was significantly, positively related to attachment anxiety (*β* = .26) and other-oriented perfectionism (*β* = .22) among mothers.

## Discussion

The presented study was devoted to identification of potential determinants of parental identity development among mothers. The results confirmed significant hypothesized relationships between both attachment and perfectionism of mothers with their parental identity but not all hypothesized relationships turned out to be significant and, in general, the observed relationships were quite weak. As expected, attachment anxiety and avoidance were positively correlated with reconsideration of parental commitment. However, only attachment anxiety was significantly, negatively correlated with commitment, and in the case of in-depth exploration, the links with attachment were insignificant. In line with the meta-analysis of Årseth et al. (2009), those observations support the notion that attachment development and identity development might by two developmental processes that influence each other only to a limited extent. As regards perfectionism, it was expected that self-oriented perfectionism will be positively related to identity commitment and in-depth exploration, and negatively to reconsideration of commitment and that in the cases of other-oriented perfectionism and socially prescribed perfectionism results will be quite the opposite. However, it turned out that parental identity commitment was not related to perfectionism at all, and that in-depth exploration was only weakly, positively correlated with self-oriented perfectionism. The hypothesized relationships were observed to the highest extent in the case of reconsideration of commitment, which, as expected, was positively correlated to attachment avoidance, attachment anxiety, other-oriented perfectionism, and socially prescribed perfectionism. As a result, we could suggest here that both adult attachment and perfectionism are related rather to reconsidering of parental commitment that was made earlier than to the processes of forming parental identity commitment (commitment and in-depth exploration). However, as indicated by the results of regression analysis, some of those correlates seem to have especially important, specific relationships with parental identity.

The adult attachment dimensions and perfectionism were also related to each other. Attachment anxiety and avoidance were positively related especially to socially prescribed perfectionism that is in line with other studies (Chen, Hewitt, & Flett, 2015). Mothers who were higher on attachment avoidance and anxiety were characterized by a stronger belief that people who are important to them expect them to be flawless and perfect (socially-prescribed perfectionism). It is also in accordance with the ideas presented by Hewitt, Flett, and Mikail (2017) and with the results of the studies on the relationships between insecure attachment and maladaptive perfectionism (Wei et al., 2004; Ko, Hewitt, Cox, Flett, & Chen, 2019). The present study is the first that clearly shows that both of these characteristics are also related to the sense of parental identity among mothers.

### Adult attachment and parental identity formation

Adult romantic attachment, although related to early childhood experiences, is characterized by its own dynamic and development (Hazan & Shaver, 1987). People experiencing strong attachment anxiety and/or avoiding closeness in relationships create intimate relationships that give them less satisfaction, perceive low support from their partner, and the relationship itself is more often a source of stress and discomfort (Mikulincer & Shaver, 2007). What is more, attachment avoidance and anxiety negatively influence functioning as a parent being related to non-optimal parenting styles and non-responsiveness to a child’s needs (Frost, Lahart, Rosenblate, 1991; Gong, Fletcher, & Bolin, 2015; Smith et al., 2017).

The obtained results suggest that from a perspective of development of the mother’s parental identity, attachment patterns are less related to the process of commitment to the parental role and to the process of gathering information about the role of a parent, being rather associated with the process of considering whether the decision to become a parent was not a mistake and with higher regret about being a parent (reconsideration of commitment). As a result, we can suppose that attachment patterns may influence some final stages of the mother’s parental identity development in the form of accepting the role of a parent as a part of the self. Mothers who are less securely attached to a partner seem to be more likely to regret parenthood. It is in line with the results of Crocetti, Rubini, and Meeus (2008) who showed, in turn, that distrust for parents, an important component of insecure attachment, makes it difficult for adolescents to form their identities. The results of the regression analysis suggest that the dimension of attachment anxiety seems to be of particular importance. Similarly, Macek et al. (2015) showed that romantic attachment anxiety is negatively related to clarity of self. Previous studies have also shown that trait-anxiety (Spielberger et al., 1983) disturbs the development of identity (Crocetti et al., 2009), also in the parental domain (Piotrowski, 2018). In the present study, however, it was shown for the first time that this specific type of anxiety is also uniquely associated with the mother’s parental identity.

Mothers fearing abandonment by their partner and feeling that they are of little importance to him, to a lesser extent identified with the role of a parent, and perceived parenting as less satisfactory and giving less hope for the future (commitment). They were also to a greater extent disappointed with motherhood and believed that the lack of a child would make their lives more interesting and better (reconsideration of commitment). Mothers who are concerned about the availability of a partner may perceive parenthood as a source of danger. When a mother is looking for great closeness but sees her partner as insensitive and inaccessible (regardless of whether it is an objective fact or only her subjective perception), she may be afraid that if he leaves, she will be left alone with the child and the responsibility for care will rest only on her. Research confirms that such fears lead to less satisfaction with parenthood (Nelson, Kushlev, & Lyubomirsky, 2014). This might be the reason why attachment avoidance, related to the feeling of being self-sufficient and denying the importance of close relationships (Mikulincer & Shaver, 2007), may do not translate into difficulties with parental identity formation to such extent.

### Perfectionism and parental identity formation

Self-oriented perfectionism manifests itself in setting very high expectations and striving for perfection in everything that is done (Hewitt & Flett, 1991). Luyckx, Soenens et al. (2008) and Piotrowski (2019) have observed that high expectations towards oneself, while controlling perfectionistic fears and doubts, generally favor undertaking identity commitment and identification with them and stimulate the exploration process by motivating reflection on one’s own identity. In the presented study, it turned out that the pursuit of perfection in mothers translates to a certain degree to their greater interest in children and parenting, a more intensive search for information regarding upbringing and considering what kind of parent one wants to be (in-depth exploration). This may be a sign of the self-oriented perfectionistic mothers’ strive to becoming a perfect parent, however, the effect of this motivation does not seem to have a strong, specific impact on parental identity. In general, it must be stressed that the expected link between self-oriented perfectionism and parental identity was smaller than anticipated. Based on the results of Luyckx et al. (2008) and Piotrowski (2019) we could expect stronger relationships. However, Luyckx and colleagues and Piotrowski studied the future plans identity domain (future plans clarity) and not the parental one. It may be suggested though that parental identity might be less influenced by the individual’s striving for perfection than the domain of future plans identity. The relative independence of identity domains (e.g. vocational identity, relational identity etc.) has been supported by recent studies (Vosylis, Erentaitė, & Crocetti, 2018) and also parental identity was suggested as being specific to some extent (Piotrowski, 2018). I think that this issue needs more time and more studies to be solved.

Another dimension of perfectionism that was expected to have some impact on the formation of parental identity is socially prescribed perfectionism (the belief that other people expect an individual to be perfect), which positively correlated with the reconsideration of commitment. Socially-prescribed perfectionism is perceived as one of the core maladaptive aspects of perfectionism (Stoeber & Otto, 2006) which is a risk factor for depression, anxiety disorders, eating disorders, obsessive-compulsive disorder (Limburg, Watson, Hagger, & Egan, 2016). Luyckx, Soenens et al. (2008) and Piotrowski (2019) observed that characteristics of perfectionism similar to socially-prescribed perfectionism (they studied concern over mistakes and doubts about actions that along with socially-prescribed perfectionism form the higher-order maladaptive perfectionism factor; Stoeber & Otto, 2006) may also disrupt identity development processes. The results obtained in the present study suggesting that socially-prescribed perfectionism correlates positively with higher reconsideration of commitment in the parental domain is thus in line with the previous studies. What was not expected were so weak relationships between socially-prescribed perfectionism and identity commitment and in-depth exploration. Maybe this aspect of perfectionism does not influence the processes of commitment to the parental role but rather the further evaluation and acceptance of the role. Taking the obtained results on self-oriented and socially-prescribed perfectionism together it looks like two of the most often studied aspects of perfectionism (Stoeber & Otto, 2006) have less to do with parental identity formation that it could be expected. However, even the relationship between socially prescribed perfectionism and parental identity was not observed in the regression analysis, which suggests that it may result from the association of this form of perfectionism with other variables studied such as attachment anxiety and with the key, as it appears, other-oriented perfectionism, which turned out to be in a specific way related to parental identity.

Stoeber (2014, 2015) showed that other-oriented perfectionists perceive themselves as ideal and expect others to be perfect, get into conflicts, accuse others of not being good enough and being an obstacle on the perfectionist’s path to success. It is no surprise, therefore, that social relations created by them are full of tension and disagreement (Stoeber, 2012). Present study suggests that it is this attitude of mothers towards other people that is most clearly associated with an increased identity crisis in the parental domain. It seems that excessive expectations towards others make it especially difficult for women to develop a stable parental identity when such a person becomes a parent. For mothers characterized by increased other-oriented perfectionism, family life is probably associated with many frustrations and stress, hence the focus on alternative visions of themselves that seem to be better than the current situation of being a parent. Other studies have revealed that other-oriented perfectionism increases the likelihood of being disappointed with one’s partner (Stoeber, 2012). The result obtained shows that this characteristic may also lead to disappointment with the child and motherhood.

In sum, the obtained results suggest that attachment anxiety and other-oriented perfectionism may play particularly important roles in the process of parental identity formation after a woman becomes a mother. Because these two variables were not correlated (Table 1), they can be considered as two, largely independent, specific predictors of identity formation in the parental domain among mothers.

Additionally, the auxiliary analysis also revealed a negative correlation between mothers’ age (but not children’s age) and identity commitment suggesting that commitment in the parental domain may become weaker with the increasing age. As the youngest mothers in the study were in their early twenties and the oldest ones were close to forty this observation suggests a different approach to parenthood among mothers in different developmental periods. Younger participants were still in the emerging adulthood period which is characterized by dynamic life changes and a sense of instability (Arnett, 2000). In their case, being a mother might stabilize the sense of identity to a greater extent and, as it was observed, lead to a stronger parental identity commitment. For mothers who were in their thirties, the parental role might be less important for a general sense of identity because of their stronger involvement in other life domains such as a professional career and thus their parental identity commitment might become weaker. This interpretation is supported to some extent by the results obtained by Nelson et al. (2007) who observed that emerging adults rated role transitions (e.g. parenthood) as more important for the adult status than did their parents. However, because of a rather small sample in the present study, it was not possible to explore an issue of mother’s age and parental identity thoroughly so it is recommended to do it in the future.

### Limitations and suggestions for further research

The presented study, despite new knowledge that has been obtained, also has a number of limitations. First of all, only Polish mothers took part in the study which makes it difficult to generalize results. In further studies, it is advisable to take a closer look at the group of fathers and carry out similar research in other countries (in order to facilitate this type of research, *U-MICS: Parental Identity* have been included in Appendixes A and B). Second, the results presented are based on a single cross-sectional study and do not provide information about causal relationships. It is recommended to verify these results and to analyze the development of parental identity longitudinally. Thirdly, the obtained results also do not allow to answer the question as to the mechanism of potential influence of the attachment style and perfectionism on parental identity. Other studies suggest that both of these characteristics often affect adjustment indirectly, e.g., through the impact on experiencing negative emotions (e.g. Ashby, Rice, & Martin, 2011). Conducting further research, it is worth devoting a place to this issue as well. The presented study focused on the relationships between personality characteristics and mothers’ parental identity; in further research, it is worth focusing more on the relationship between parental identity and behavioral indicators of parenthood such as parental engagement and parenting styles.

## Conclusions

Neo-Eriksonian research on identity in recent years has focused on the analysis of dynamics of identity development in various domains: educational, professional, relational, vision of own future, financial. Researchers analyze the course of commitment in these areas, factors related to increase of exploration, and the pursuit of identity change. So far, however, not much space has been devoted to the parental identity associated with implementation of perhaps the most important social role of adulthood. Recent research on this issue (Fadjukoff et al., 2016, Piotrowski, 2018) suggest that the parental domain, although it may be distinguished by a certain specificity, should be analyzed within the same conceptual system. Young people who become parents incorporate this sphere into their own identity via commitment processes, explore this commitment, and finally, in some cases, they may feel regret, disappointment, and desire to change their identity.

The obtained results indicate two important areas worth paying attention to, from both a scientific and practical point of view: mother’s fear of abandonment by the partner and feeling of being of little importance to him, as well as the mother’s expectation of perfection from other people. Both of these characteristics, independently of one another, can probably lead to a similar effect, such as disappointment with motherhood and a dream about how good it would be to turn back time (see Donath, 2015; Piotrowski, 2018). The obtained results may also be useful for other scholars in explaining non-optimal parenting and parental psychopathology as high regret about being a parent can be an important negative predictor of parents’ involvement in childcare and their mental health.

## Additional Files

The additional files for this article can be found as follows:

10.5334/pb.492.s1 Appendix A. English language version of the U-MICS: Parental Identity.

10.5334/pb.492.s2 Appendix B. Polish language version of the U-MICS: Parental Identity.
