# Supplementary material for: How Good It Would Be to Turn Back Time: Adult Attachment and Perfectionism in Mothers and Their Relationships with the Processes of Parental Identity Formation
Source: Psychol Belg. 2020 Feb 28;60(1):55–72. doi: 10.5334/pb.492 (PMC7047756; doi:10.5334/pb.492)
Supplement: Appendix A. — English language version of the U-MICS: Parental Identity. [file pb-60-1-492-s1.pdf]

APPENDIX A. *English language version of the U-MICS: Parental Identity* (other published versions of the U-MICS can be found in the article by Crocetti, Schwartz, Fermani, and Meeus, 2010)

Instruction

Below are a number of questions about you and your parenting. In each case, place a cross in the box that most closely matches your opinion.

1	2	3	4	5
Completely untrue	Untrue	Sometimes true / sometimes untrue	True	Completely true

1. Being a parent gives me security in life
2. Being a parent gives me self-confidence
3. Being a parent makes me feel sure of myself
4. Being a parent gives me security for the future
5. Being a parent allows me to face the future with optimism
6. I try to find out a lot about my child/children
7. I often reflect on my child / children
8. I make a lot of effort to keep finding out new things about my child / children
9. I often try to find out what other people think about my child / children
10. I often talk with other people about my child / children
11. I often think it would have been better not to have had any children
12. I often think that not having a child / children would have made my life more interesting
13. In fact I believe that it would have been better for me not to have been a parent at all

Scoring:

1-5: Commitment

6-10: In-depth exploration

11-13: Reconsideration of commitment
