# Supplementary material for: How Good It Would Be to Turn Back Time: Adult Attachment and Perfectionism in Mothers and Their Relationships with the Processes of Parental Identity Formation
Source: Psychol Belg. 2020 Feb 28;60(1):55–72. doi: 10.5334/pb.492 (PMC7047756; doi:10.5334/pb.492)
Supplement: Appendix B. — Polish language version of the U-MICS: Parental Identity. [file pb-60-1-492-s2.pdf]

APPENDIX B. *Polish language version of the U-MICS: Parental Identity* (other published versions of the U-MICS can be found in the article by Crocetti, Schwartz, Fermani, and Meeus, 2010)

Instrukcja

Poniżej znajdują się stwierdzenia dotyczące ciebie i twojego rodzicielstwa. Ustosunkuj się do każdego z nich, wskazując odpowiedź która najlepiej do Ciebie pasuje.

1	2	3	4	5
Zdecydowanie nieprawda	Nieprawda	Czasami prawda / czasami nieprawda	Prawda	Zdecydowanie prawda

1. Bycie rodzicem daje mi poczucie bezpieczeństwa w życiu
2. Bycie rodzicem dodaje mi wiary w siebie.
3. Bycie rodzicem sprawia, że czuję się pewnie.
4. Bycie rodzicem zapewnia mi bezpieczną przyszłość.
5. Bycie rodzicem pozwala mi patrzeć w przyszłość z optymizmem.
6. Staram się dowiedzieć jak najwięcej o moim dziecku / moich dzieciach.
7. Często myślę o moim dziecku / moich dzieciach.
8. Wkładam wiele wysiłku, aby dowiedzieć się czegoś nowego o moim dziecku / moich dzieciach.
9. Często staram się dowiedzieć, co inni myślą o moim dziecku / moich dzieciach.
10. Często rozmawiam z innymi o moim dziecku / moich dzieciach.
11. Często myślę, że lepiej byłoby nie mieć dziecka / dzieci w ogóle.
12. Często myślę, że brak dziecka / dzieci uczyniłby moje życie bardziej ciekawym.
13. Tak naprawdę, sądzę że lepiej dla mnie byłoby gdybym nigdy nie został(-a) rodzicem.

Klucz:

1-5: Zaangażowanie

6-10: Eksploracja w głąb

11-13: Rewizja zaangażowania
